# *Canscora lucidissima*, a Chinese folk medicine, exerts anti-inflammatory activities by inhibiting the phosphorylation of ERK1/2 in LPS-activated macrophages

**DOI:** 10.1186/s12906-019-2783-2

**Published:** 2019-12-16

**Authors:** Qiao-ling Fei, Xiao-yu Zhang, Rui-juan Qi, Yun-feng Huang, Yi-xin Han, Xi-meng Li, Run-lan Cai, Yuan Gao, Yun Qi

**Affiliations:** 1 Institute of Medicinal Plant Development, Chinese Academy of Medical Sciences and Peking Union Medical College, 151 North Ma Lian Wa Road, Haidian District, Beijing, 100193 China; 2 Guangxi Institute of Chinese Medicine and Pharmaceutical Sciences, Nanning, China

**Keywords:** *Canscora lucidissima*, Lipopolysaccharide (LPS), Macrophages, ERK1/2, Endotoxemia

## Abstract

**Background:**

*Canscora lucidissima* (Levl. & Vaniot) Hand.-Mazz. (*C. lucidissima*), mainly distributed in southern China, has been shown to be effective in the treatment of inflammatory diseases. However, the underlying mechanism of its anti-inflammatory effect is not fully understood.

**Methods:**

In this study, we investigated the anti-inflammatory mechanism of ethanol extract of *C. lucidissima* (Cl-EE) in lipopolysaccharide (LPS)-induced inflammatory models. ELISA, real-time PCR, Western blot and luciferase reporter assay were used for the experiments in vitro, and ICR mouse endotoxemia model was used for in vivo test.

**Results:**

Our data showed that Cl-EE reduced the production of NO by down-regulating the mRNA and protein expression of inducible nitric oxide synthase (iNOS) in LPS-activated RAW 264.7 cells. Meanwhile, it potently decreased other proinflammatory mediators, such as TNF-α, IL-6, MCP-1 and IL-1β at the transcriptional and translational levels. Further study indicated that Cl-EE did not affect NF-κB signaling pathway but significantly suppressed the phosphorylation of ERK1/2, rather than JNK or p38. In a LPS-induced endotoxemia mouse model, a single intraperitoneal injection of Cl-EE (75–300 mg/kg) could lower circulatory TNF-α, IL-6 and MCP-1 levels.

**Conclusions:**

Collectively, our results indicated that Cl-EE suppressed the phosphorylation level of ERK1/2 thus reducing the transcription and translation of inflammatory genes, thereby exerted anti-inflammatory activity. This study reveals the anti-inflammatory mechanism of *C. lucidissima* and may provide an effective treatment option for a variety of inflammatory diseases.

## Background

As an important pathological process, inflammation occurred in many diseases in response to tissue injuries or host defenses against pathogenic microorganism [1]. In the inflammatory process, key events involve the release of pro-inflammatory cytokines and/or chemokines, etc. [2]. As the defense cells, macrophages play a crucial role in killing pathogen microbes and resulting in activating an arsenal of anti-microbial effectors, and consequently triggering the inflammatory cascade [3]. Lipopolysaccharide (LPS) is a component of the outer membrane of Gram-negative bacteria. It can be regarded as a pathogen-associated molecular pattern and can be recognized by the membrane receptor TLR4 on macrophages, and subsequently leading to the activation of NF-κB and AP-1 and initiating the transcription of downstream inflammatory cytokines [4].

The IκB kinase (IKK) complex, consisting of IKKα, IKKβ and the regulatory subunit NEMO, plays a vital role in myeloid cells. It is well-documented that IKKβ, rather than IKKα [5, 6], can phosphorylate IκBα and lead to its ubiquitination and degradation, thus freeing NF-κB p65 into cell nucleus. Besides, IKK complex activation also causes the polyubiquitination and degradation of NF-κB1 p105 [7], which releases TPL-2 from NF-κB1 p105 inhibition and allows TPL-2 to phosphorylate its substrate MEK [8]. And activated MEK phosphorylates ERK1/2 and activates AP-1 signaling. Moreover, the activated NF-κB or AP-1 can also translocate into the nucleus and promote the transcription of proinflammatory cytokines, such as inducible nitric oxide synthase (iNOS), TNF-α, IL-1β, IL-6 and MCP-1 etc. Although the release of these mediators is a protective response of the immune system to microbial invasion, their excessive production can aggravate inflammation and even threaten life in severe cases. Thus, effective anti-inflammatory agents are indispensable to suppress these excessive cytokines [9].

**Fig. 1:**
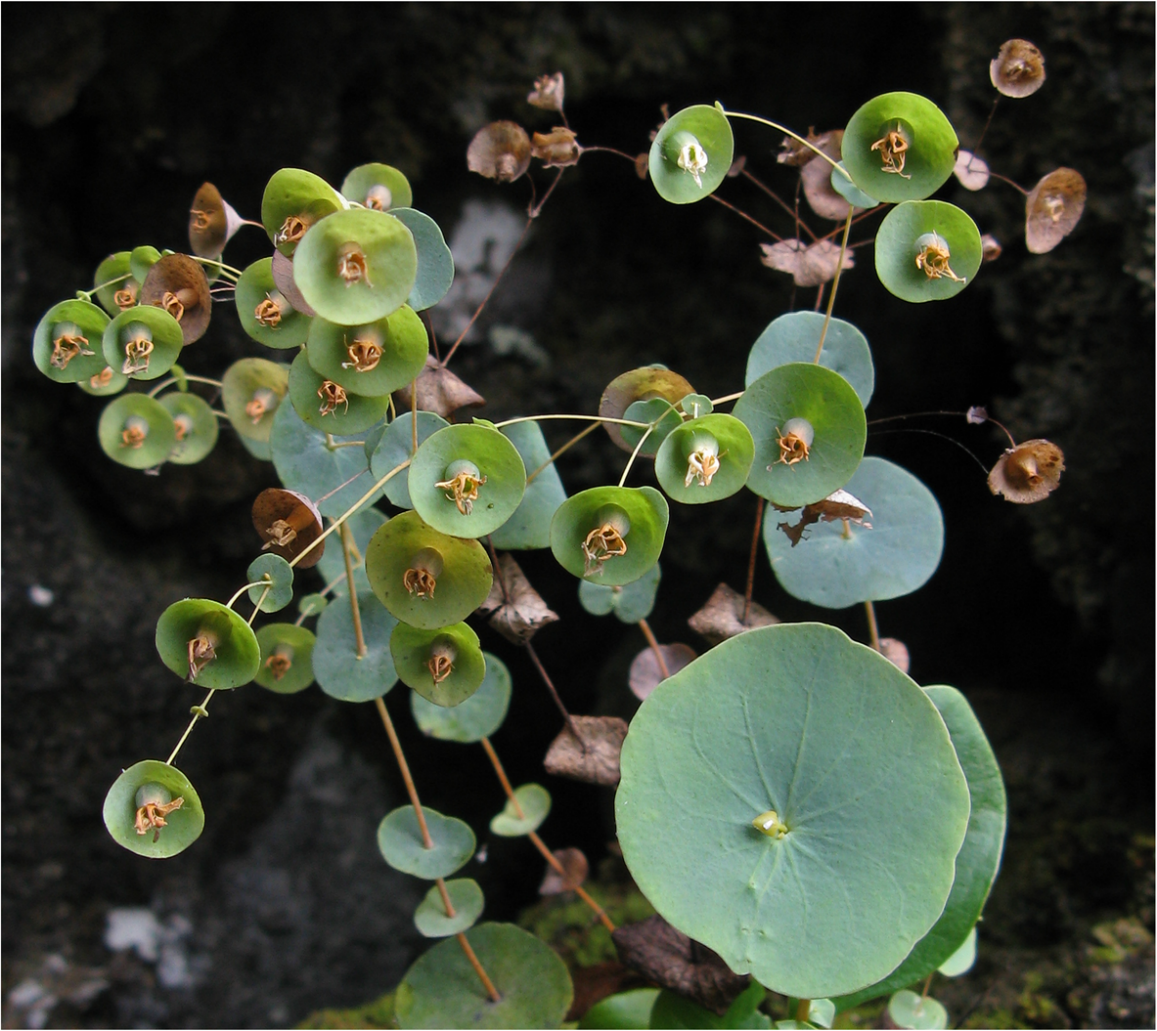
The original plant of *C. lucidissimia* obtained from Guangxi province

Gentianaceae herb *Canscora lucidissima* (Levl. & Vaniot) Hand.-Mazz. (*C. lucidissima*; Fig. 1) mainly distributes in Guangxi and Guizhou provinces of China. It is widely used by the local residents (Zhuang nationality) for the treatment of inflammation-related diseases, such as acute mastitis, impetigo, trauma injury, wasp and snake bites, etc. [10]. Xanthones, a class of constituents in *C. lucidissima*, were found to ameliorate acute inflammation, including ear/paw edema swelling and peritoneal capillary permeability [11]. Moreover, congeneric herb *Canscora decussate* (Roxb.) Schult also exhibits considerably anti-inflammatory activity in vitro and in vivo [12]. Nevertheless, no study focused on the anti-inflammatory effect of *C. lucidissima*. The present study aims to evaluate the effects of the ethanol extract of *C. lucidissima* (Cl-EE) on inflammation in LPS-activated macrophages and the corresponding in vivo model (endotoxemia mice) and further explore the underlying mechanism.

## Methods

### Materials and reagents

DMEM and FBS were produced by Gibco BRL (Grand Island, NY, USA). Mouse TNF-α, IL-6 and MCP-1 ELISA kits were obtained from Biolegend (San Diego, CA, USA). Mouse IL-1β ELISA kit was purchased from Excell Technology Co. (Shanghai, China). Antibody against iNOS was obtained from Santa Cruz Biotechnology, Inc. (Santa Cruz, California, USA). Antibodies against JNK, ERK1/2, p38, phospho-JNK (p-JNK), phospho-ERK1/2 (p-ERK1/2), phospho-p38 (p-p38), phospho-IκBα (p-IκBα) and NF-κB p65 were obtained from Cell Signaling Technology (Danvers, CO, USA). Antibodies against β-actin were obtained from Cwbiotech Co. (Beijing, China). Horseradish peroxidase-conjugated anti-rabbit or anti-mouse IgG secondary antibodies and Histone H3 polyclonal antibody were obtained from Abclonal Biotechonology Co. Ltd. (Wuhan, Hubei, China). TRIzol® Reagent was from Thermo Fisher (Van Allen Way, Carlsbad CA). M-MuLV First Strand cDNA Synthesis Kit and oligonucleotide primers were from Sangon Biotech (Shanghai, China). KAPA SYBR® FAST Universal 2X qPCR Master Mix kit was obtained from Kapa Biosystems Pty Ltd. (Salt River Cape Town, South Africa). The plasmid for pNFκB-TA-luc and luciferase assay system were from Beyotime Institute of Biotechnology (Haimen, Jiangsu, China). The 3-(4,5-dimethylthiazol-2-yl)-2,5-diphenyl tetrazolium bromide (MTT), L-NG-Nitroarginine Methyl Ester (L-NAME) and LPS were from Sigma-Aldrich (St. Louis, MO, USA). All other reagents were of analytical grade.

### Animals

ICR and C57BL/6 mice (male, 18–20 g) were obtained from Vital River Experimental Animal Services (Beijing, China) and housed in a SPF laboratory under standard temperature (22 °C–24 °C) and humidity (45–65%) conditions with a 12 h light/dark cycle and standard pallet diet and water ad libitum. Animal experiments were carried out according to the National Institutes of Health Guide for Care and Use of Laboratory Animals and approved by the Institutional Animal Care and Use Committee (IACUC), Institute of Medicinal Plant Development (IMPLAD) of Chinese Academy of Medical Sciences (CAMS) [SYXK (Beijing) 2007–0020]. Anesthetic drugs and all other necessary measures were used to reduce animal suffering during the experimental procedures.

### Preparation of the plant extract

The herb of *C. lucidissima* (Fig. 1) from Guangxi Province of China was collected in June 18th 2018. The plant materials were authenticated by Prof. Yun-feng Huang according to their morphological characteristics. A voucher specimen was deposited in the Herbarium of Guangxi Institute of Chinese Medicine & Pharmaceutical Sciences. Air-dried whole herb of *C. lucidissima* (100 g) was extracted by an ethanol-water (85:15, v/v, 2 L) solution for 2 h. The ethanol solvent was removed by rotary evaporator and the thoroughly dried *C. lucidissima* extract was stored at − 20 °C with the yield of 28.7%.

### Cell isolation, culture and treatment

The murine macrophage RAW264.7 cell line was obtained from American Type Culture Collection (ATCC, Rockville, MD, USA). Mouse bone marrow-derived macrophages (BMDMs) were obtained from femurs and tibiae of C57BL/6 mice after cervical dislocation and differentiated in 10% macrophage colony-stimulating factor conditioned media for 5–8 days before use [13]. All cells were cultured in DMEM supplemented with 10% FBS, penicillin G (100 units/mL) and streptomycin (100 mg/mL) in a humidified incubator with 5% CO_2_ at 37 °C.

### Cell viability assay

MTT assay was used for the measurement of the effect of Cl-EE on cell viability [14]. Macrophages were seeded in a 96-well plate at a density of 4 × 10^5^ cells per well. After treatment with different concentrations of Cl-EE (25, 50, 100, 200 and 400 μg/mL) for 20 h, 0.5% MTT was added for a further 4 h. Then, DMSO (100 μL) was added into each well and the optical density at 540 nm was measured by a microplate reader (Thermo Fisher Scientific, USA).

### Measurement of NO production

Supernatant NO production was measured by Griess method [15]. RAW264.7 cells were seeded in a 96-well plate at a density of 4 × 10^5^ cells per well and pretreated with Cl-EE at different concentrations (25, 50 and 100 μg/mL) for 1 h. LPS (10 ng/mL) was added to the medium for a further incubation. Twenty-four hours later, nitrite production was measured by mixing 100 μL of supernatant and 100 μL of Griess reagent [0.1% (w/v) N-(1-naphthyl)-ethylenediamine and 1% (w/v) sulfanilamide in 5% (v/v) phosphoric acid]. Optical density at 540 nm was measured by a microplate reader_._

### Measurement of iNOS activity

The effect of Cl-EE on the iNOS activity was measured as we previously described [16]. RAW264.7 cells were incubated with LPS (10 ng/mL). Twelve hours later, supernatant LPS was removed by washing three times. In this context, the change of supernatant NO level is attributed to the change of iNOS enzymatic activity. The LPS-stimulated cells were harvested and plated in a 48-well plate at a density of 8 × 10^5^ cells per well and incubated with or without Cl-EE for another 12 h. The iNOS activity was expressed as the nitrite level in the culture medium. A nonselective NOS inhibitor L-NAME was used as a positive control.

### Measurement of inflammatory cytokines (TNF-α, IL-6, MCP-1 and IL-1β) in the culture medium

RAW264.7 cells were seeded at a density of 4 × 10^5^ cells per well in 96-well plates. After the cells were pretreated with Cl-EE at different concentrations for 1 h, 10 ng/mL LPS was added into the medium for another 24 h. Supernatant was collected for determining TNF-α, IL-6 and MCP-1.

Since too low IL-1β production in the supernatant of LPS-activated RAW264.7 cells, BMDMs and higher LPS concentration (40 ng/mL) were used instead for IL-1β detection. Supernatant cytokines were measured by using ELISA kits according to the manufacturer’s instructions.

### RNA extraction and quantitative real-time PCR (RT-PCR)

RT-PCR assay was performed as previously described [17]. RAW264.7 macrophages were seeded in 6-well plates at a density of 1.2 × 10^7^ cells per well overnight. The cells were pretreated with Cl-EE for 1 h and then exposed to LPS (10 ng/mL) for a further 4 h. Total RNA was extracted by using TRIzol and RT-PCR was performed by using a KAPA SYBR® FAST Universal 2X qPCR Master Mix kit. The cycling conditions were as follows: 95 °C for 20 s, 40 cycles of 95 °C for 15 s and 60 °C for 20 s. The mRNA levels of iNOS, TNF-α, MCP-1, IL-1β, and IL-6 were normalized to GAPDH, a stable housekeeping gene. The primer information used in this study was listed in Table 1.

**Table 1:** Sequences of primers used in the RT-PCR analysis

Targets	Direction	Primer sequences
iNOS	F	5’-CTC AGC CCA ACA ATA CAA G-3’
	R	5’-CTA CAG TTC CGA GCG TCA-3’
IL-6	F	5’-CTG CAA GAG ACT TCC ATC CAG-3’
	R	5’-AGT GGT ATA GAC AGG TCT GTT GG-3’
MCP-1	F	5’-GCC CCA CTC ACC TGC TGC TAC T-3’
	R	5’-CCT GCT GCT GGT GAT CCT CTT GT-3’
IL-1β	F	5’-GCA ACT GTT CCT GAA CTC AAC T-3’
	R	5’-ATC TTT TGG GGT CCG TCA ACT-3’
TNF-α	F	5’- GCC TAT GTC TCA GCC TCT T-3’
	R	5’- GGT TGA CTT TCT CCT GGT AT-3’
GAPDH	F	5’-GGT TGT CTC CTG CGA CTT CA-3’
	R	5’-TGG TCC AGG GTT TCT TAC TCC-3’

*F* forward, *R* reverse

### Luciferase reporter assay

RAW264.7 macrophages stably transfected with pNFκB-TA-luc were seeded in a 24-well plate at a density of 2.5 × 10^6^ cells per well overnight. The cells were pre-incubated with different concentrations of Cl-EE for 1 h and then exposed to LPS (10 ng/mL) for a further 4 h. The cells were lysed and the luciferase activity was measured using a luciferase assay kit.

### Western blot analysis

The Western blot assay was performed as we previously described [15]. RAW264.7 macrophages were pretreated with Cl-EE at the indicated concentrations for 1 h and then exposed to LPS for different time (ERK1/2/p-ERK1/2, JNK/p-JNK, p38/p-p38, 15 min; IκBα/p-IκBα, NF-κB p65/Histone, 30 min; iNOS, 24 h). Proteins in nucleus or cytoplasm were extracted for the assay. The blots were visualized using enhanced chemiluminescence (Applygen, Beijing, China) and data were analyzed using the Gel Doc EQ System (Bio-Rad, Hercules, USA).

### Mouse endotoxemia model

The in vivo anti-inflammatory effect of Cl-EE was investigated in the mouse endotoxemia model [18]. Forty male ICR mice were randomly divided into five groups (*n* = 8 per group): normal control (NC) group, model group (1 mg/kg LPS alone) and three Cl-EE treatment groups (1 mg/kg LPS + Cl-EE). Mice were intraperitoneally injected with sterile saline or Cl-EE (75, 150 and 300 mg/kg). Thirty minutes later, LPS (1 mg/kg) was intravenously injected to the mice except NC group. Three hours later, the mice were anesthetized by inhalation of isoflurane and the blood samples were collected from the orbital venous plexus. Finally, mice were euthanized by cervical dislocation. The levels of TNF-α, IL-6 and MCP-1 in serum were measured using ELISA kits.

### Statistical analysis

All data were expressed as mean ± standard deviation and were analyzed using the one-way ANOVA. The statistical significance was determined by Student’s *t*-test for comparisons between two groups. Differences were considered to be significant when *P* < 0.05.

## Results

### Cl-EE decreases LPS-induced supernatant NO without cytotoxicity

**Fig. 2:**
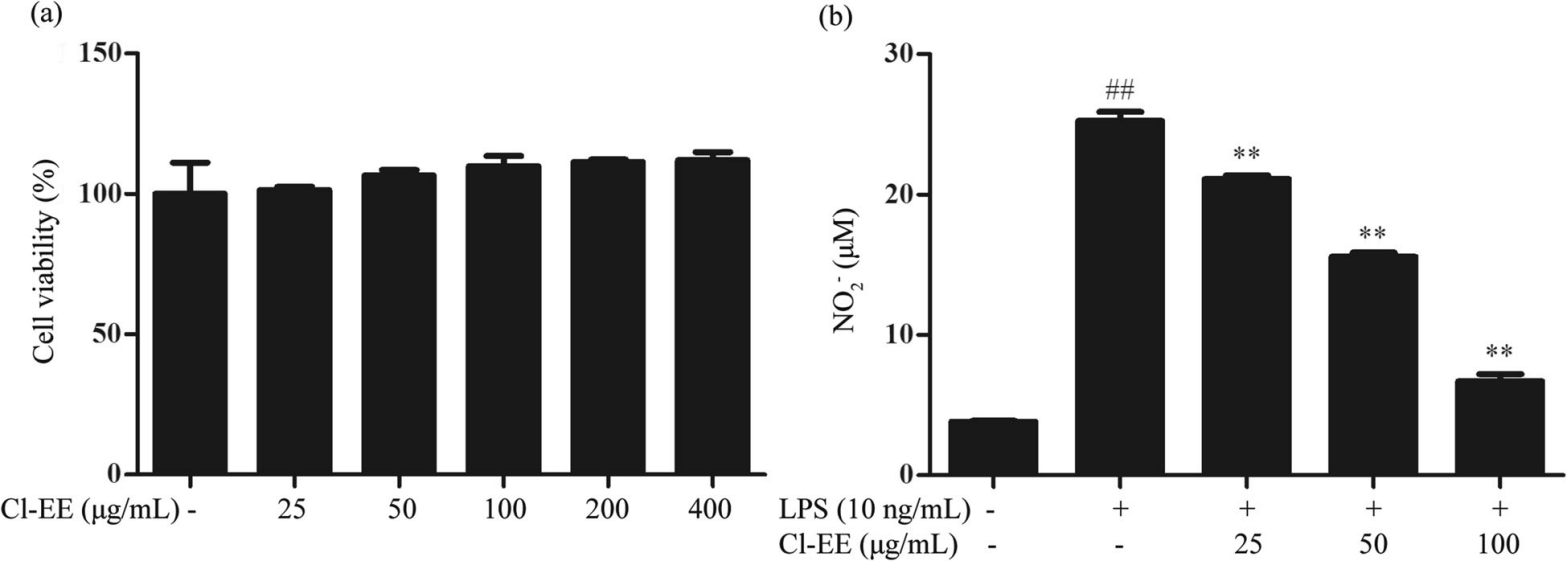
Effects of Cl-EE on cell viability and NO production in LPS-activated RAW 264.7 macrophages. **a** Effect of Cl-EE on the cell viability. RAW264.7 cells were treated with various concentrations of Cl-EE for 24 h and the cell viability was measured by MTT assay. **b** Effect of Cl-EE on LPS-induced NO production. RAW264.7 cells were pretreated with Cl-EE at the indicated concentrations for 1 h and then exposed to LPS (10 ng/mL) for 24 h. The nitrite levels in the medium were measured by the Griess reaction. ^##^*P* < 0.01 versus normal control (NC) group; ^**^P < 0.01 versus LPS alone

Cytotoxic assay showed that Cl-EE did not affect the cell viability up to 400 μg/mL (Fig. 2a and Additional file 1: Table S1). Thus, all subsequent in vitro experiments were conducted at nontoxic concentrations (25-100 μg/mL). Because a small diffusible molecule NO is a well-known marker of inflammatory response [19], we first measured the effect of Cl-EE on supernatant NO production in LPS-stimulated RAW 264.7 macrophages. As shown in Fig. 2b and Additional file 1: Table S1, LPS treatment caused a robust increase of supernatant NO, while Cl-EE potently decreased the LPS-induced NO production in a concentration-dependent manner.

### Cl-EE suppresses LPS-induced iNOS mRNA and protein expression

**Fig. 3:**
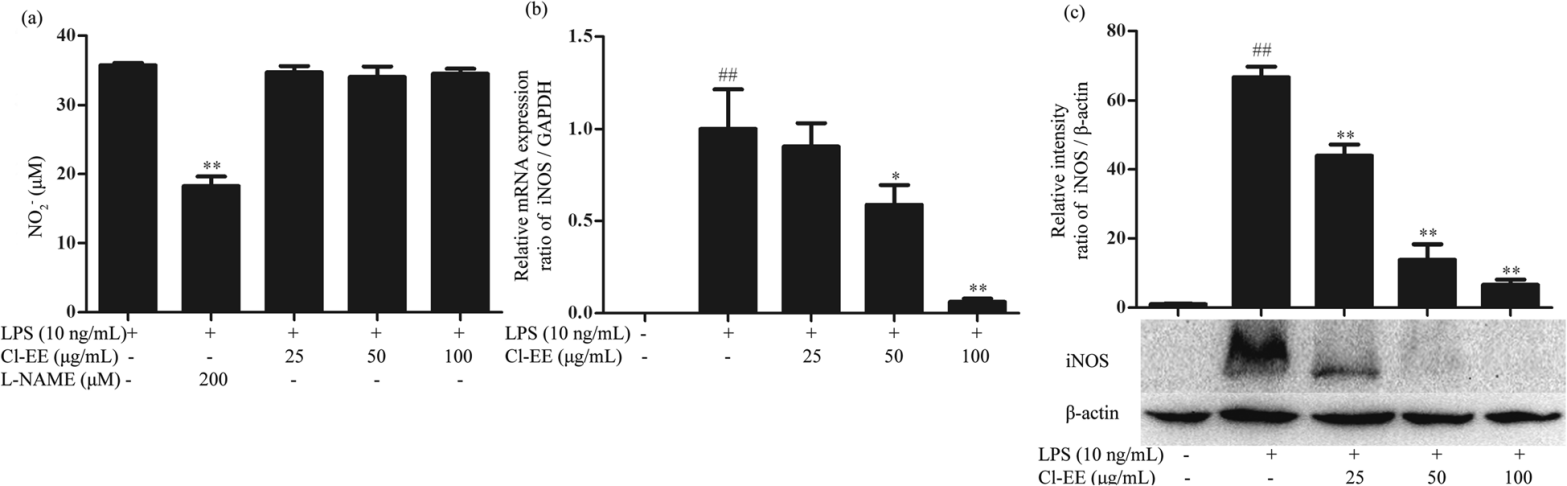
Effect of Cl-EE on iNOS in LPS-activated RAW264.7 cells. **a** Effect of Cl-EE on iNOS enzyme activity. RAW264.7 cells were pretreated with LPS (10 ng/mL) for 12 h and then exposed to Cl-EE at the indicated concentrations for another 12 h in the absence of LPS. The nitrite levels in the medium were measured by Griess method. **b** Effect of Cl-EE on iNOS mRNA expression. RAW264.7 cells were pretreated with the indicated concentrations of Cl-EE for 1 h and then exposed to LPS (10 ng/mL) for 4 h. The mRNA level of iNOS was determined by RT-PCR analysis. **c** Effect of Cl-EE on iNOS protein level. RAW264.7 cells were pretreated with Cl-EE at the indicated concentrations for 1 h and then exposed to LPS (10 ng/mL) for 24 h. Cellular proteins were extracted and the translation level of iNOS was determined by Western blot analysis. ^##^*P* < 0.01 versus NC group; ^*^P < 0.05 and ^**^P < 0.01 versus LPS alone

As NO is generated by iNOS in the activated macrophages, we next investigated whether the inhibitory effect of Cl-EE on supernatant NO was attributed to the down-regulation of iNOS activity or expression in LPS-stimulated RAW264.7 cells. As shown in Fig. 3a and Additional file 2: Table S2, the positive control L-NAME significantly inhibited iNOS activity, but Cl-EE could not. We subsequently investigated the effects of Cl-EE on the iNOS mRNA and protein levels. As shown in Fig. 3b and c and Additional file 2: Table S2, treatment with Cl-EE concentration-dependently inhibited the LPS-elevated iNOS expression at both transcriptional and translational levels. Thus, Cl-EE decreased supernatant NO in LPS-activated RAW 264.7 cells via the suppression of iNOS mRNA and protein expression, rather than inhibiting its activity.

### Cl-EE reduces LPS-elevated TNF-α, IL-6, MCP-1 and IL-1β expression

**Fig. 4:**
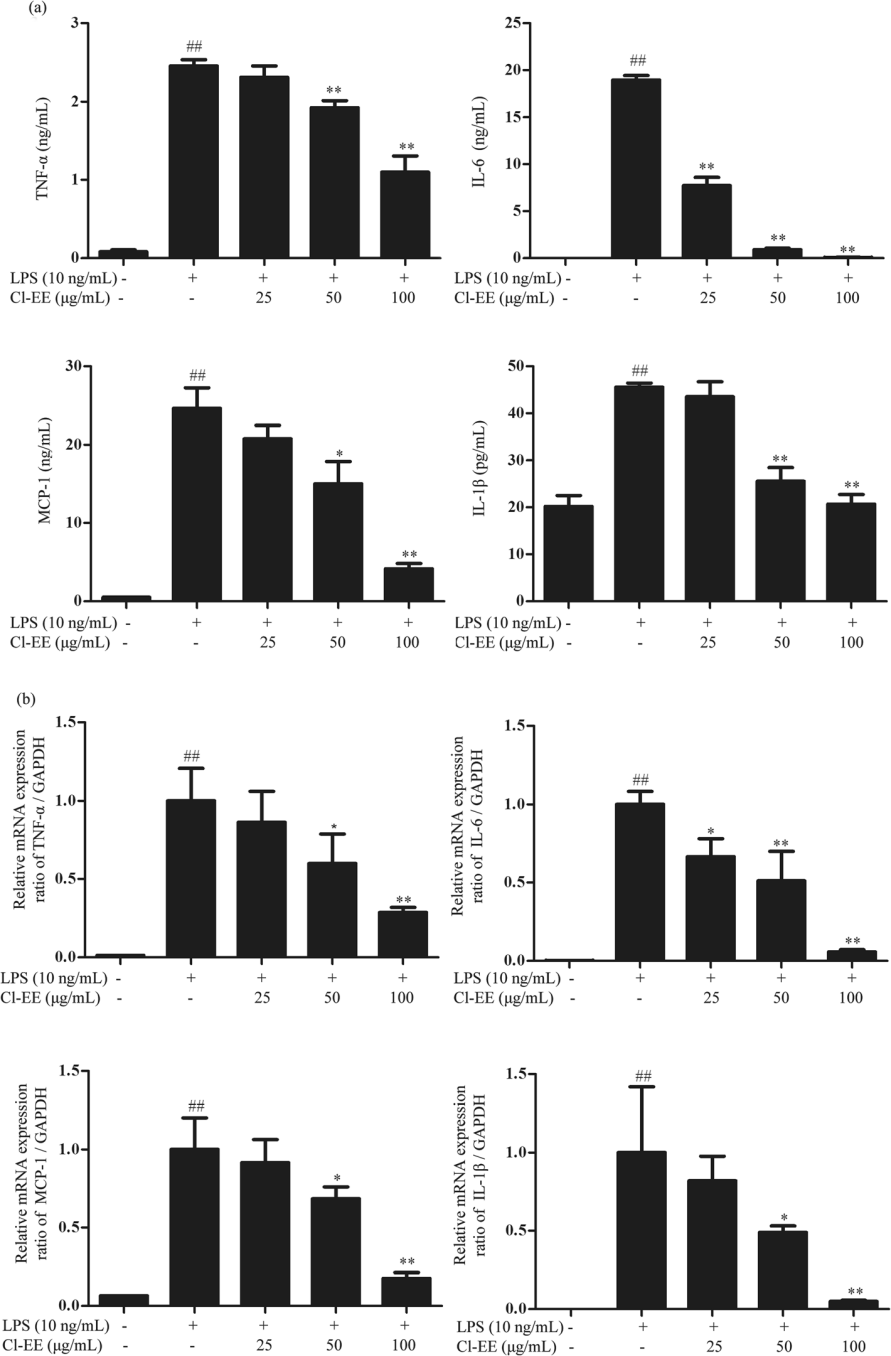
Effects of Cl-EE on proinflammatory cytokines at transcriptional and translational levels in LPS-activated macrophages. **a** Effects of Cl-EE on the supernatant proinflammatory cytokines in LPS-activated macrophages. RAW264.7 cells were pretreated with Cl-EE for 1 h at the indicated concentrations and then exposed to LPS (10 ng/mL) for TNF-α, IL-6 and MCP-1 determination. BMDMs were pretreated with Cl-EE for 1 h and then exposed to LPS (40 ng/mL) for IL-1β assay. The production of TNF-α, IL-6, MCP-1 and IL-1β in the supernatant were determined by ELISA. **b** Effects of Cl-EE on the mRNA levels of the proinflammatory cytokines in LPS-activated RAW264.7 cells. Cells were pretreated with Cl-EE at the indicated concentrations for 1 h and then exposed to LPS (10 ng/mL) for 4 h. The mRNA expression of TNF-α, IL-6, MCP-1, and IL-1β were determined by RT-PCR analysis. ^##^*P* < 0.01 versus NC group; ^*^P < 0.05 and ^**^P < 0.01 versus LPS alone

Apart from NO, other proinflammatory cytokines, such as TNF-α, MCP-1, IL-6 and IL-1β, also play crucial roles in inflammatory processes. Our data showed that Cl-EE concentration-dependently suppressed LPS-elevated supernatant TNF-α, MCP-1, IL-6 in RAW 264.7 cells. It also decreased the level of supernatant IL-1β in LPS-primed BMDMs (Fig. 4a and Additional file 3: Table S3). Next, we investigated the effect of Cl-EE on their mRNA levels using RT-PCR. Consistently, Cl-EE also could suppress LPS-induced TNF-α, MCP-1, IL-6 and IL-1β mRNA transcription (Fig. 4b and Additional file 3: Table S3).

### Cl-EE has no effect on LPS-activated NF-κB signaling pathway

**Fig. 5:**
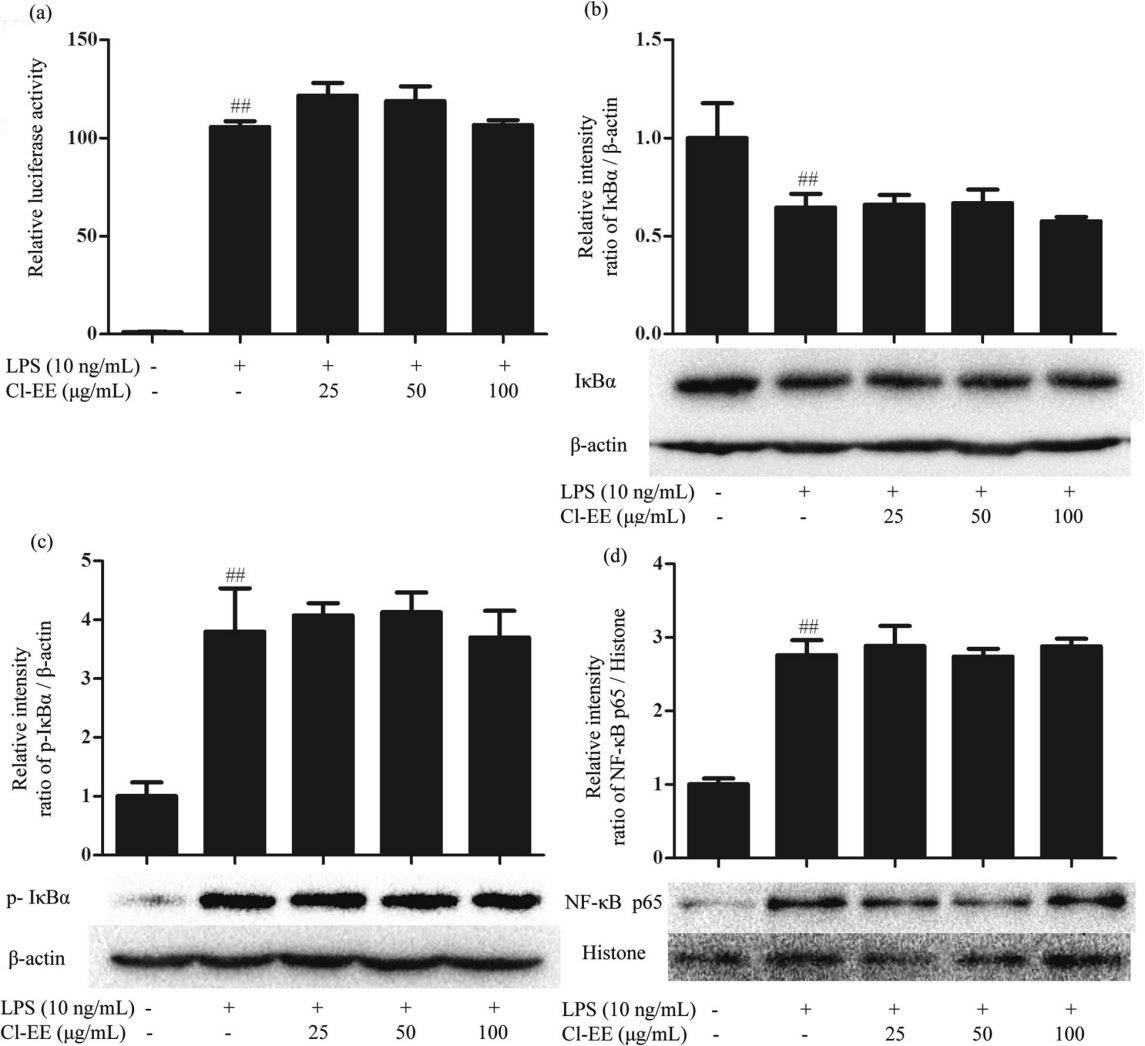
Effects of Cl-EE on LPS-induced activation of NF-κB signaling pathway. **a** Effect of Cl-EE on LPS-induced NF-κB luciferase action. RAW264.7 cells transfected with the luciferase reporter pNFκB-TA-luc were pretreated with Cl-EE at the indicated concentrations for 1 h and then exposed to LPS (10 ng/mL) for 4 h. NF-κB activity was detected by luciferase assay system. **b**-**d** Effects of Cl-EE on cytosol IκBα, cytosol p-IκBα and the nuclear translocation of NF-κB p65 in LPS-activated RAW264.7 cells. The cells were pretreated with Cl-EE at the indicated concentrations for 1 h and then were exposed to LPS (10 ng/mL) for 30 min. Cytosolic and nuclear proteins were extracted for Western blot analysis. ^##^*P* < 0.01 versus NC group

As a classical dimeric transcription factor in inflammatory processes, NF-κB can regulate the gene transcription of many proinflammatory mediators [20]. Thus, we investigated the effect of Cl-EE on the NF-κB signaling pathway by using luciferase assay system. The obtained results indicated that Cl-EE did not inhibit NF-κB-dependent transcriptional activity (Fig. 5a and Additional file 4: Table S4). Consistently, Cl-EE little affected the phosphoralation of IκBα and the subsequent nucleus translocation of p65 (Fig. 5b-d and Additional file 4: Table S4).

### Cl-EE suppresses LPS-induced phosphorylation of ERK1/2

**Fig. 6:**
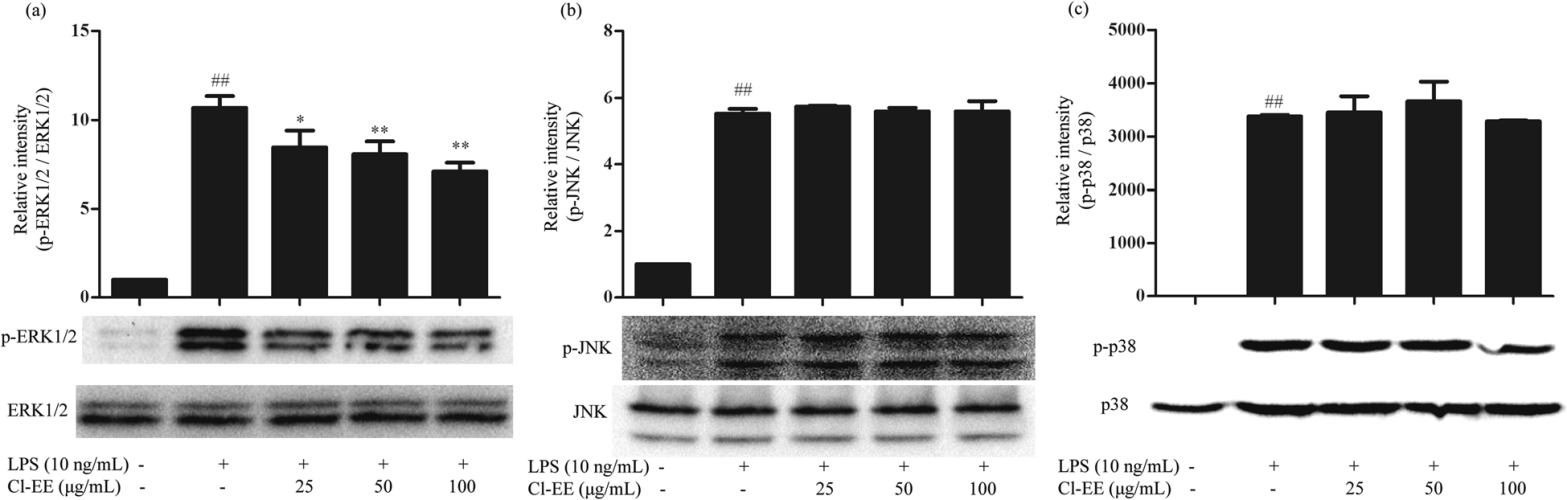
Effects of Cl-EE on LPS-induced phosphorylation of MAPKs. **a** ERK1/2, **b** JNK, and **c** p38. RAW264.7 cells were pretreated with Cl-EE at the indicated concentrations for 1 h and then exposed to LPS (10 ng/mL) for 15 min. Total proteins were extracted for Western blot analysis. ^##^*P* < 0.01 versus NC group; ^*^P < 0.05 and ^**^P < 0.01 versus LPS alone

Since Cl-EE had little action on the NF-κB signaling pathway, we next focused on another important signaling pathway-MAPKs, which mainly consisted of three components (ERK1/2, JNK and p38). The results showed that LPS markedly elevated the phosphorylation levels of ERK1/2, JNK and p38, while treatment with Cl-EE significantly reduced p-ERK1/2 in a concentration-dependent manner without affecting the levels of p-JNK and p-p38 (Fig. 6 and Additional file 5: Table S5).

### Cl-EE inhibits LPS-elevated circulatory inflammatory cytokines in vivo

**Fig. 7:**
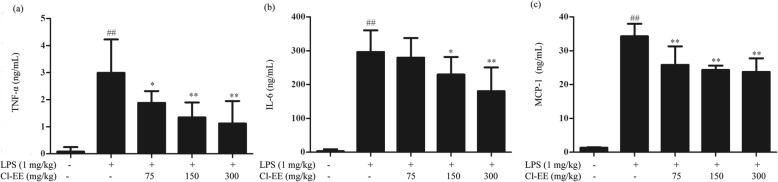
Effects of Cl-EE on circulatory proinflammatory cytokines levels in LPS-induced endotoxemia mice (*n* = 8). Mice received (75, 150 and 300 mg/kg, i.p.) Cl-EE 0.5 h before LPS stimulation (1 mg/kg, i.v.). Three hours after LPS challenge, whole blood was collected. The levels of **a** TNF-α, **b** IL-6 and **c** MCP-1 in serum were measured by ELISA. ^##^*P* < 0.01 versus NC group; ^*^P < 0.05 and ^**^P < 0.01 versus LPS alone

The in vivo anti-inflammatory effect of Cl-EE was evaluated using a mouse endotoxemia model. As shown in Fig. 7 and Additional file 6: Table S6, LPS administration (1 mg/kg, i.v.) markedly increased serum TNF-α, IL-6 and MCP-1 levels compared with the NC group, while pretreatment with Cl-EE (75, 150 and 300 mg/kg, i.p.) decreased the production of these inflammatory cytokines dose-dependently.

## Discussion

The herb of *C. lucidissima* has been widely used to treat inflammatory diseases as Zhuang folk medicine in south of China, especially in Guangxi and Guizhou provinces. Although *C. lucidissima* is considered to be a potent anti-inflammatory herb, its underlying mechanism remains unknown. To this end, we explored the anti-inflammatory activities and the mechanism of *C. lucidissima* using the LPS-stimulated macrophages and mouse endotoxemia model. To our knowledge, the traditional extract way of *C. lucidissima* includes water decoction (high-polar molecular-rich), vinum (both high- and low-polar molecule-rich) and oil (low-polar molecule-rich). In a limited number of chemical researches, low-polar xanthones were found to have definite anti-inflammatory activity [11]. Together with our preliminary experiment, we extracted *C. lucidissima* by 85% ethanol to enrich the main anti-inflammatory ingredients in the present study. The obtained extract (Cl-EE) was firstly evaluated the cytotoxic effect on macrophages using MTT assay. The data showed that Cl-EE did not affect cell viability up to 400 μg/mL (Fig. 2a and Additional file 1: Table S1). Thus, we selected 25, 50, 100 μg/mL of Cl-EE in the in vitro experiments.

Diffusible micromolecule NO plays an important role in maintaining the homeostasis of the immune system, while excessive NO is associated with the initiation of inflammatory diseases [21]. In the present study, supernatant NO was potently decreased by Cl-EE in a concentration-dependent manner in LPS-activated RAW264.7 cells (Fig. 2b and Additional file 1: Table S1). During the inflammatory processes, large amounts of NO are generated by iNOS in macrophages [22]. Our data showed that Cl-EE down-regulated iNOS transcriptional and translational levels without inhibiting its activity (Fig. 3 and Additional file 2: Table S2).

Apart from NO, other proinflammatory cytokines, such as TNF-α, IL-6, MCP-1 and IL-1β, also play essential roles in the inflammatory processes. TNF-α is an important cytotoxic mediator that can lead to tissue destruction, cytotoxicity, and acute inflammation. IL-6 is regarded as an endogenous mediator of LPS-induced fever [23] and IL-1β can initiate and enhance the inflammatory response [24]. MCP-1, a CC-chemokine, triggers inflammation process including leukocyte attachment to endothelium and recruitment to inflammation site [25]. Our data showed that Cl-EE effectively suppressed the levels of these inflammatory cytokines in the supernatant of LPS-activated macrophages (Fig. 4a and Additional file 3: Table S3), showing a multifaceted action. Consistently, their mRNA levels were also down-regulated by Cl-EE (Fig. 4b and Additional file 3: Table S3).

NF-κB is a classical signaling pathway in the regulation of the production of proinflammatory mediators, which depends on the phosphorylation and degradation of IκB [26]. As one member of NF-κB family, p65 has a C-terminal transactivation domain that has intrinsic ability to activate transcription. In this study, the elevated levels of p-IκBα (cytosol) and p65 (nuclear) by LPS were not suppressed by Cl-EE (Fig. 5b-d and Additional file 4: Table S4). Consistently, in the luciferase reporter gene assay, Cl-EE did not affect the NF-κB transcriptional activity (Fig. 5a and Additional file 4: Table S4), suggesting that the anti-inflammatory effect of Cl-EE was not attributed to inhibiting the activation of NF-κB signal.

AP-1, another conventional transcription factor, acts downstream of MAPKs which consists of three components, JNK, ERK1/2 and p38. Activated MAPKs are able to phosphorylate their respective substrate proteins, thus initiate proinflammatory cytokines transcription [27]. In our study, Cl-EE selectively diminished LPS-induced phosphorylation of ERK1/2 but did not affect the phosphorylation levels of JNK and p38 (Fig. 6 and Additional file 5: Table S5).

Endotoxemia is a systemic response to serious infection and yields a poor prognosis when it is associated with organ dysfunction, hypoperfusion, or hypotension [28]. It is also associated with cytokines storm characterized by the upregulation of proinflammatory cytokines [29]. Therefore, we evaluated whether Cl-EE could attenuate LPS-caused endotoxemia. In line with our findings in vitro, Cl-EE indeed decreased LPS-induced circulatory inflammatory mediators release (TNF-α, IL-6 and MCP-1) (Fig. 7 and Additional file 6: Table S6).

## Conclusions

In summary, our findings demonstrated that the anti-inflammatory activity of Cl-EE was attributed to attenuating the phosphorylation of ERK1/2, thereby suppressing iNOS, TNF-α, MCP-1, IL-6 and IL-1β at transcriptional and translational levels in LPS-activated macrophages. Meanwhile, Cl-EE could lower LPS-elevated circulatory inflammatory cytokines in vivo.

## Supplementary information

**Additional file 1: Table S1.** Raw data for Fig. 2.

**Additional file 2: Table S2** Raw data for Fig. 3.

**Additional file 3: Table S3** Raw data for Fig. 4.

**Additional file 4: Table S4** Raw data for Fig. 5.

**Additional file 5: Table S5** Raw data for Fig. 6.

**Additional file 6: Table S6** Raw data for Fig. 7.

## Data Availability

The datasets used and analysed during the current study are available from the corresponding author on reasonable request.

## Abbreviations

BMDMs: Bone marrow-derived macrophages
*C. lucidissima*: *Canscora lucidissima*
Cl-EE: Ethanol extract of *C. lucidissima*
IKK: Inhibitor of κB kinase
iNOS: inducible nitric oxide synthase
L-NAME: L-NG-nitroarginine methyl ester
LPS: Lipopolysaccharide
MTT: 3-(4,5-dimethylthiazol-2-yl)-2,5-diphenyl tetrazolium bromide
NC: Normal control
p-ERK1/2: phospho-ERK1/2
p-IκBα: phospho-IκBα
p-JNK: phospho-JNK
p-p38: phospho-p38. RT-PCR: real-time PCR.
