# Supplementary material for: Canscora lucidissima, a Chinese folk medicine, exerts anti-inflammatory activities by inhibiting the phosphorylation of ERK1/2 in LPS-activated macrophages
Source: BMC Complement Altern Med. 2019 Dec 16;19:371. doi: 10.1186/s12906-019-2783-2 (PMC6916437; doi:10.1186/s12906-019-2783-2)
Supplement: Supplementary file 1 — Additional file 1: Table S1. Raw data for Fig. 2. [file 12906_2019_2783_MOESM1_ESM.pdf]

**Table S1** Raw data for figure 2.

a. Effect of Cl-EE on the cell viability in LPS-activated RAW 264.7 cells.

| Cl-EE (μg/ml) | Mean | SD | P |
|---|---|---|---|
| - | 100.000 | 11.200 | - |
| 25 | 101.410 | 1.109 | 0.848 |
| 50 | 106.536 | 2.076 | 0.419 |
| 100 | 109.933 | 3.612 | 0.261 |
| 200 | 111.471 | 0.872 | 0.217 |
| 400 | 112.015 | 2.884 | 0.199 |

b. Effect of Cl-EE on LPS-induced NO production in LPS-activated RAW 264.7 cells.

| LPS (ng/ml) | Cl-EE (μg/ml) | Mean | SD | P |
|---|---|---|---|---|
| 0 | 0 | 3.838 | 0.085 | - |
| 10 | 0 | 25.262 | 0.642 | < 0.001 |
| 10 | 25 | 21.103 | 0.296 | 0.003 |
| 10 | 50 | 15.575 | 0.324 | < 0.001 |
| 10 | 100 | 6.715 | 0.522 | < 0.001 |
