# Supplementary material for: Canscora lucidissima, a Chinese folk medicine, exerts anti-inflammatory activities by inhibiting the phosphorylation of ERK1/2 in LPS-activated macrophages
Source: BMC Complement Altern Med. 2019 Dec 16;19:371. doi: 10.1186/s12906-019-2783-2 (PMC6916437; doi:10.1186/s12906-019-2783-2)
Supplement: Supplementary file 2 — Additional file 2: Table S2 Raw data for Fig. 3. [file 12906_2019_2783_MOESM2_ESM.pdf]

Table S2 Raw data for figure 3.

a. Effect of Cl-EE on iNOS enzyme activity in LPS-activated RAW264.7 cells.

LPS (ng/ml)	L-NAME (μm)	Cl-EE (μg/ml)	Mean	SD	P
10	0	0	647.452	6.452	-
10	200	0	317.613	26.613	< 0.001
10	0	25	628.097	17.742	0.150
10	0	50	615.194	29.032	0.133
10	0	100	624.065	13.710	0.056

b. Effect of Cl-EE on iNOS mRNA expression in LPS-activated RAW264.7 cells.

LPS (ng/ml)	Cl-EE (μg/ml)	Mean	SD	P
0	0	0.000	0.000	-
10	0	1.000	0.216	< 0.001
10	25	0.905	0.127	0.476
10	50	0.589	0.107	0.014
10	100	0.061	0.018	< 0.001

c. Effect of Cl-EE on iNOS protein level in LPS-activated RAW264.7 cells.

LPS (ng/ml)	Cl-EE (μg/ml)	Mean	SD	P
0	0	1.000	0.000	-
10	0	65.715	6.595	< 0.001
10	25	41.030	2.196	0.003
10	50	13.351	3.536	< 0.001
10	100	6.260	1.345	< 0.001
