# Supplementary material for: Canscora lucidissima, a Chinese folk medicine, exerts anti-inflammatory activities by inhibiting the phosphorylation of ERK1/2 in LPS-activated macrophages
Source: BMC Complement Altern Med. 2019 Dec 16;19:371. doi: 10.1186/s12906-019-2783-2 (PMC6916437; doi:10.1186/s12906-019-2783-2)
Supplement: Supplementary file 3 — Additional file 3: Table S3 Raw data for Fig. 4. [file 12906_2019_2783_MOESM3_ESM.pdf]

Table S3 Raw data for figure 4.

a. Effects of Cl-EE on the supernatant proinflammatory cytokines in LPS-activated macrophages.

(1) Effects of Cl-EE on the supernatant TNF-α in LPS-activated RAW264.7 cells.

LPS (ng/ml)	Cl-EE (μg/ml)	Mean	SD	P
0	0	0.080	0.025	-
10	0	2.453	0.082	< 0.001
10	25	2.308	0.147	0.209
10	50	1.925	0.090	0.002
10	100	1.099	0.207	0.003

(2) Effects of Cl-EE on the supernatant IL-6 in LPS-activated RAW264.7 cells.

LPS (ng/ml)	Cl-EE (μg/ml)	Mean	SD	P
0	0	0.002	0.001	-
10	0	18.950	0.468	< 0.001
10	25	7.710	0.891	< 0.001
10	50	0.878	0.150	< 0.001
10	100	0.054	0.012	< 0.001

(3) Effects of Cl-EE on the supernatant MCP-1 in LPS-activated RAW264.7 cells.

LPS (ng/ml)	Cl-EE (μg/ml)	Mean	SD	P
0	0	0.477	0.025	-
10	0	24.649	2.626	< 0.001
10	25	20.758	1.695	0.097
10	50	15.008	2.865	0.012
10	100	4.133	0.671	< 0.001

(4) Effects of Cl-EE on the supernatant IL-1β in LPS-activated BMDMs.

LPS (ng/ml)	Cl-EE (μg/ml)	Mean	SD	P
0	0	20.179	2.350	-
10	0	45.564	0.888	0.001
10	25	43.513	3.203	0.384
10	50	25.564	2.912	0.004
10	100	20.692	2.035	< 0.001

b. Effects of Cl-EE on the mRNA levels of the proinflammatory cytokines in LPS-activated RAW264.7 cells.

(1) Effects of Cl-EE on the mRNA levels of TNF-α in LPS-activated RAW264.7 cells.

LPS (ng/ml)	Cl-EE (μg/ml)	Mean	SD	P
0	0	0.013	0.000	-
10	0	1.000	0.208	< 0.001
10	25	0.863	0.197	0.418
10	50	0.600	0.188	0.047
10	100	0.286	0.031	< 0.001

(2) Effects of Cl-EE on the mRNA levels of IL-6 in LPS-activated RAW264.7 cells.

LPS (ng/ml)	Cl-EE (μg/ml)	Mean	SD	P
0	0	0.003	0.000	-
10	0	1.000	0.083	< 0.001
10	25	0.666	0.114	0.011
10	50	0.511	0.188	0.001
10	100	0.058	0.014	< 0.001

(3) Effects of Cl-EE on the mRNA levels of MCP-1 in LPS-activated RAW264.7 cells.

LPS (ng/ml)	Cl-EE (μg/ml)	Mean	SD	P
0	0	0.066	0.000	-
10	0	1.000	0.202	< 0.001
10	25	0.916	0.147	0.571
10	50	0.684	0.077	0.027
10	100	0.177	0.036	< 0.001

(4) Effects of Cl-EE on the mRNA levels of IL-1β in LPS-activated RAW264.7 cells.

LPS (ng/ml)	Cl-EE (μg/ml)	Mean	SD	P
0	0	0.001	0.000	-
10	0	1.000	0.419	0.003
10	25	0.819	0.157	0.675
10	50	0.489	0.042	0.033
10	100	0.049	0.008	< 0.001
