# Supplementary material for: Canscora lucidissima, a Chinese folk medicine, exerts anti-inflammatory activities by inhibiting the phosphorylation of ERK1/2 in LPS-activated macrophages
Source: BMC Complement Altern Med. 2019 Dec 16;19:371. doi: 10.1186/s12906-019-2783-2 (PMC6916437; doi:10.1186/s12906-019-2783-2)
Supplement: Supplementary file 4 — Additional file 4: Table S4 Raw data for Fig. 5. [file 12906_2019_2783_MOESM4_ESM.pdf]

Table S4 Raw data for figure 5.

a. Effect of Cl-EE on LPS-induced NF-κB luciferase action in LPS-activated RAW264.7 cells.

LPS (ng/ml)	Cl-EE (μg/ml)	Mean	SD	P
0	0	1.000	0.145	-
10	0	105.642	3.008	< 0.001
10	25	121.691	6.468	0.057
10	50	118.887	7.591	0.079
10	100	106.695	2.484	0.665

b. Effects of Cl-EE on cytosol IκBα in LPS-activated RAW264.7 cells.

LPS (ng/ml)	Cl-EE (μg/ml)	Mean	SD	P
0	0	1.000	0.178	-
10	0	0.645	0.070	0.010
10	25	0.660	0.050	0.703
10	50	0.668	0.069	0.609
10	100	0.571	0.014	0.063

c. Effects of Cl-EE on cytosol p-IκBα in LPS-activated RAW264.7 cells.

LPS (ng/ml)	Cl-EE (μg/ml)	Mean	SD	P
0	0	1.000	0.236	-
10	0	3.798	0.738	0.003
10	25	4.071	0.210	0.571
10	50	4.127	0.337	0.521
10	100	3.699	0.452	0.853

d. Effects of Cl-EE on the nuclear translocation of NF-κB p65 in LPS-activated RAW264.7 cells.

LPS (ng/ml)	Cl-EE (μg/ml)	Mean	SD	P
0	0	1.000	0.037	-
10	0	2.758	0.093	< 0.001
10	25	2.883	0.124	0.558
10	50	2.739	0.048	0.888
10	100	2.879	0.046	0.409
