# Supplementary material for: Canscora lucidissima, a Chinese folk medicine, exerts anti-inflammatory activities by inhibiting the phosphorylation of ERK1/2 in LPS-activated macrophages
Source: BMC Complement Altern Med. 2019 Dec 16;19:371. doi: 10.1186/s12906-019-2783-2 (PMC6916437; doi:10.1186/s12906-019-2783-2)
Supplement: Supplementary file 5 — Additional file 5: Table S5 Raw data for Fig. 6. [file 12906_2019_2783_MOESM5_ESM.pdf]

Table S5 Raw data for figure 6.

a. Effects of Cl-EE on LPS-induced phosphorylation of ERK1/2 in LPS-activated RAW264.7 cells.

LPS (ng/ml)	Cl-EE (μg/ml)	Mean	SD	P
0	0	1.000	0.000	-
10	0	10.694	0.661	< 0.001
10	25	8.457	0.953	0.029
10	50	8.071	0.725	0.009
10	100	7.094	0.499	0.002

b. Effects of Cl-EE on LPS-induced phosphorylation of JNK in LPS-activated RAW264.7 cells.

LPS (ng/ml)	Cl-EE (μg/ml)	Mean	SD	P
0	0	1.000	0.000	-
10	0	5.533	0.140	< 0.001
10	25	5.739	0.023	0.066
10	50	5.596	0.110	0.573
10	100	5.596	0.306	0.762

c. Effects of Cl-EE on LPS-induced phosphorylation of p38 in LPS-activated RAW264.7 cells.

LPS (ng/ml)	Cl-EE (μg/ml)	Mean	SD	P
0	0	1.000	0.000	-
10	0	3376	30.005	< 0.001
10	25	3453	301.558	0.824
10	50	3661	371.228	0.525
10	100	3287	14.406	0.116
