# Supplementary material for: Canscora lucidissima, a Chinese folk medicine, exerts anti-inflammatory activities by inhibiting the phosphorylation of ERK1/2 in LPS-activated macrophages
Source: BMC Complement Altern Med. 2019 Dec 16;19:371. doi: 10.1186/s12906-019-2783-2 (PMC6916437; doi:10.1186/s12906-019-2783-2)
Supplement: Supplementary file 6 — Additional file 6: Table S6 Raw data for Fig. 7. [file 12906_2019_2783_MOESM6_ESM.pdf]

Table S6 Raw data for Figure 7.

a. Effects of Cl-EE on TNF-α in the serum of LPS-induced endotoxemia mice.

LPS (mg/kg)	Cl-EE (mg/kg)	Mean	SD	P
0	0	0.082	0.168	-
1	0	2.992	1.235	< 0.001
1	75	1.887	0.430	0.032
1	150	1.346	0.553	0.004
1	300	1.119	0.835	0.003

b. Effects of Cl-EE on IL-6 in the serum of LPS-induced endotoxemia mice.

LPS (mg/kg)	Cl-EE (mg/kg)	Mean	SD	P
0	0	3.313	5.641	-
1	0	296.625	63.911	< 0.001
1	75	279.750	57.933	0.589
1	150	229.938	52.044	0.038
1	300	180.393	70.594	0.005

c. Effects of Cl-EE on MCP-1 in the serum of LPS-induced endotoxemia mice.

LPS (mg/kg)	Cl-EE (mg/kg)	Mean	SD	P
0	0	1.335	0.117	-
1	0	34.279	3.711	< 0.001
1	75	25.860	5.450	0.003
1	150	24.310	1.303	< 0.001
1	300	23.754	4.016	< 0.001
